# The urologist’s learning curve of “in-bore” magnetic resonance-guided prostate biopsy

**DOI:** 10.1186/s12894-021-00936-y

**Published:** 2021-12-06

**Authors:** Barak Rosenzweig, Tomer Drori, Orit Raz, Gil Goldinger, Gadi Shlomai, Dorit E. Zilberman, Moshe Shechtman, Jacob Ramon, Zohar A. Dotan, Orith Portnoy

**Affiliations:** 1 Department of Urology, Chaim Sheba Medical Center, 5262080 Ramat Gan, Israel; 2 The Sackler Faculty of Medicine, Tel Aviv University, Tel Aviv, Israel; 3 The Talpiot Medical Leadership Program, Chaim Sheba Medical Center, Tel Hashomer, Ramat Gan, Israel; 4 Assuta Ashdod University Hospital, Ashdod, Israel; 5 Department of Internal Medicine D and the Hypertension Unit, Chaim Sheba Medical Center, Tel Hashomer, Ramat Gan, Israel; 6 Department of Anesthesiology, Chaim Sheba Medical Center, Ramat Gan, Israel; 7 Department of Diagnostic Imaging, Chaim Sheba Medical Center, Ramat Gan, Israel

**Keywords:** Prostate MRI, In-bore MRI, Prostate biopsy, Learning curve

## Abstract

**Background:**

The combination of multi-parametric MRI to locate and define suspected lesions together with their being targeted by an MRI-guided prostate biopsy has succeeded in increasing the detection rate of clinically significant disease and lowering the detection rate of non-significant prostate cancer. In this work we investigate the urologist’s learning curve of in-bore MRI-guided prostate biopsy which is considered to be a superior biopsy technique.

**Materials and methods:**

Following Helsinki approval by The Chaim Sheba Medical Center ethics committee in accordance with The Sheba Medical Center institutional guidelines (5366-28-SMC) we retrospectively reviewed 110 IB-MRGpBs performed from 6/2016 to 1/2019 in a single tertiary center. All patients had a prostate multi-parametric MRI finding of at least 1 target lesion (prostate imaging reporting and data system [PI-RADS] score ≥ 3). We analyzed biopsy duration and clinically significant prostate cancer detection of targeted sampling in 2 groups of 55 patients each, once by a urologist highly trained in IB-MRGpBs and again by a urologist untrained in IB-MRGpBs. These two parameters were compared according to operating urologist and chronologic order.

**Results:**

The patients’ median age was 68 years (interquartile range 62–72). The mean prostate-specific antigen level and prostate size were 8.6 ± 9.1 ng/d and 53 ± 27 cc, respectively. The mean number of target lesions was 1.47 ± 0.6. Baseline parameters did not differ significantly between the 2 urologists’ cohorts. Overall detection rates of clinically significant prostate cancer were 19%, 55%, and 69% for PI-RADS 3, 4 and 5, respectively. Clinically significant cancer detection rates did not differ significantly along the timeline or between the 2 urologists. The average duration of IB-MRGpB targeted sampling was 28 ± 15.8 min, correlating with the number of target lesions (*p* < 0.0001), and independent of the urologist’s expertise. Eighteen cases defined the cutoff for the procedure duration learning curve (*p* < 0.05).

**Conclusions:**

Our data suggest a very short learning curve for IB-MRGpB-targeted sampling duration, and that clinically significant cancer detection rates are not influenced by the learning curve of this technique.

## Introduction

Magnetic resonance-guided prostate biopsy (MRGpB) is considered superior to transrectal ultrasound (TRUS)-guided biopsy, and multi-parametric magnetic resonance imaging (mpMRI) is now regarded as a leading tool in diagnosing clinically significant prostate cancer [1–3]. The combination of mpMRI to locate and define suspected lesions together with their being targeted by an MRI-guided prostate biopsy (MRGpB) has succeeded in increasing the detection rate of clinically significant disease and lowering the detection rate of non-significant prostate cancer [1]. Incorporating MRI data with the biopsy technique can be accomplished by means of several methods. The “MR/TRUS fusion” approach uses software-based registration platforms which overlay the TRUS images with the MRI, allowing the physician to target lesions seen only on the mpMRI at the corresponding location in real-time sonographic imaging. In the “in-bore” approach, following an initial diagnostic mpMRI, the patient undergoes a biopsy within the bore of the magnet at a later date [4, 5]. These high-end technologies incorporate multidisciplinary efforts, and may therefore suggest a long learning curve and possible slow adaptation of biopsy techniques whose applications are limited by the number of trained personnel. Indeed, prostate MRI reading itself has been associated with a significant learning curve [6–8]. Similarly, a TRUS biopsy is also reportedly subject to some learning curve, and that its operator serves as an independent predictor of prostate cancer detection [9, 10]. The MR/TRUS fusion approach also bears a significant learning curve on both the individual physician as well as on the institution [11–13].

The in-bore MRI-guided prostate biopsy (IB-MRGpB) is considered by some to be a superior biopsy technique [14]. To the best of our knowledge, the learning curve for IB-MRGpB has not been reported before, and that is the aim of the current study.

## Materials and methods

### Methods and study population

Following Helsinki approval by The Chaim Sheba Medical Center ethics committee in accordance with The Sheba Medical Center institutional guidelines (5366-28-SMC) and waiver of informed consent, we retrospectively reviewed 110 consecutive IB-MRGpBs performed from June 2016 to January 2019 in a single tertiary center. The patients had been referred to our institution by their urologist or general practitioner due to elevated PSA serum levels and/or abnormal digital rectal examination, and following an mpMRI finding of at least one target lesion, which is defined as a score ≥ 3 on the prostate imaging reporting and data system (PI-RADS v.2).

### MRI analysis and biopsy technique

The referring institutions had carried out the mpMRI studies by means of 1.5 T or 3 T MRI scanners, and some of them had applied an endorectal coil and a variety of imaging protocols. All of those studies included multiplanar high-resolution T2, diffusion-weighted and dynamic contrast enhancement series. All of the scans and original readings were reviewed by a single expert radiologist who had more than 8 years’ experience in prostate mpMRI reading. Only patients with lesions suspected as clinically significant cancer (i.e., PI-RADS score ≥ 3) were sent to IB-MRGpB. The same radiologist attended all subsequent biopsy procedures, and reviewed the previous diagnostic and MRI images taken real-time during guided biopsy. The IB-MRGpBs were carried out with 3 T MRI scanners and external coil application.

The IB-MRGpB patients were placed in a prone position and administered general anesthesia. A transrectal probe (DynaTRIM; Philips, USA) containing an MR “visible” gel was positioned against the apex of the prostate and attached externally to a manual biopsy device (DynaTRIM; Philips, USA). Axial and sagittal T2-weighted images were obtained to visualize the prostate and identify the target lesion. Diffusion-weighted series were used at the radiologist’s discretion. A dedicated software package was used for device tracking and target localization (DynaCAD; Philips, USA) as described elsewhere [15]. Suspected clinically significant target lesions that were detected by MRI were sampled first, followed by 12-core template systematic prostate sampling when applicable.

The biopsies were performed by 2 senior urologists. The first urologist was trained and highly experienced in IB-MRGpBs (> 250 cases). The second urologist had no prior experience in performing IB-MRGpBs. Following training comprised of observation and a stepwise-guided hands-on approach with 8 cases, the second urologist performed the subsequent biopsies independently. All of the IB-MRGpBs sessions included in this study were then performed by one of these 2 urologists. With the exception of the urologist, the same team that was comprised of a radiologist, anesthesiologist, nurse, and technicians participated in the procedures. The patients were monitored in the recovery room for 1–2 h post-biopsy and discharged home following the anesthesiologist’s evaluation.

Biopsy specimens were processed by routine pathologic fixation with formalin solution and evaluated by a single dedicated uropathologist with > 20 years of experience. Cancer cells retrieved in the IB-MRGpB specimens were used as the reference standard to determine the biopsy result. Clinically significant disease was defined as a biopsy Gleason score of ≥ 7 (International Society of Urological Pathology, ISUP ≥ 2).

### Learning curve evaluation

Evaluation of the IB-MRGpB learning curve consisted of assessing biopsy duration and histology results according to the precision of the PI-RADS score (a “hit”). Procedure duration was defined as the time that elapsed from the first MRI scan at the time of performing the IB-MRGpB until the last target lesion sampling. We analyzed target lesion characteristics on the MRI, and assessed their effect on procedure duration according to number of lesions, lesion size, and use of diffusion MRI studies at the time of biopsy (as a surrogate for difficulty in identifying the target lesion). We divided the cohort into thirds per chronological order, and evaluated the duration of time needed to sample target lesions accordingly, i.e., comparing target sampling time at the first, second, and last thirds of patients, controlling for lesion characteristics. We then compared procedure time between the expert and novice urologists.

Since all of the MRIs before and during biopsies were read by a single radiologist, we used percentage of clinical significant disease diagnosis (ISUP ≥ 2) per PI-RADS score as surrogate for IB-MRGpB precision (“hit”). We also analyzed the effect of chronological order, lesion size as well as the urologist’s background (trained vs. untrained) on IB-MRGpB precision.

### Statistical analysis

We applied 2-sample t tests, Levene's test, and ANOVA to compare the “trained” and the “untrained” urologists’ performances. A *p* value < 0.05 was considered statistically significant. Time analysis was by logarithmic conversion, and multiple comparison testing by Benjamini–Hochberg correction. All tests were calculated using SPSS (version 25.0, IBM Corporation).

## Results

**Fig. 1:**
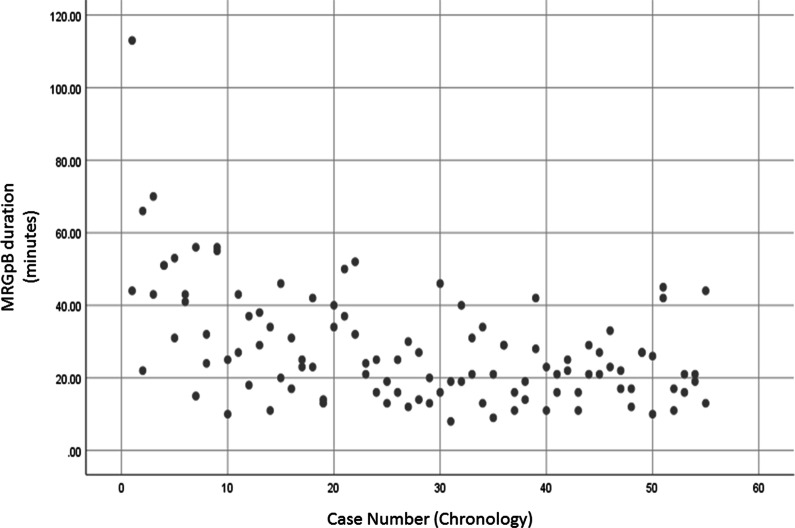
IB-MRGpB time (minutes) showing a gradual decrease in procedure time in chronological order of procedure performance. The total procedure time is given, representing 55 procedures for each of the urologists (n = 110)

**Fig. 2:**
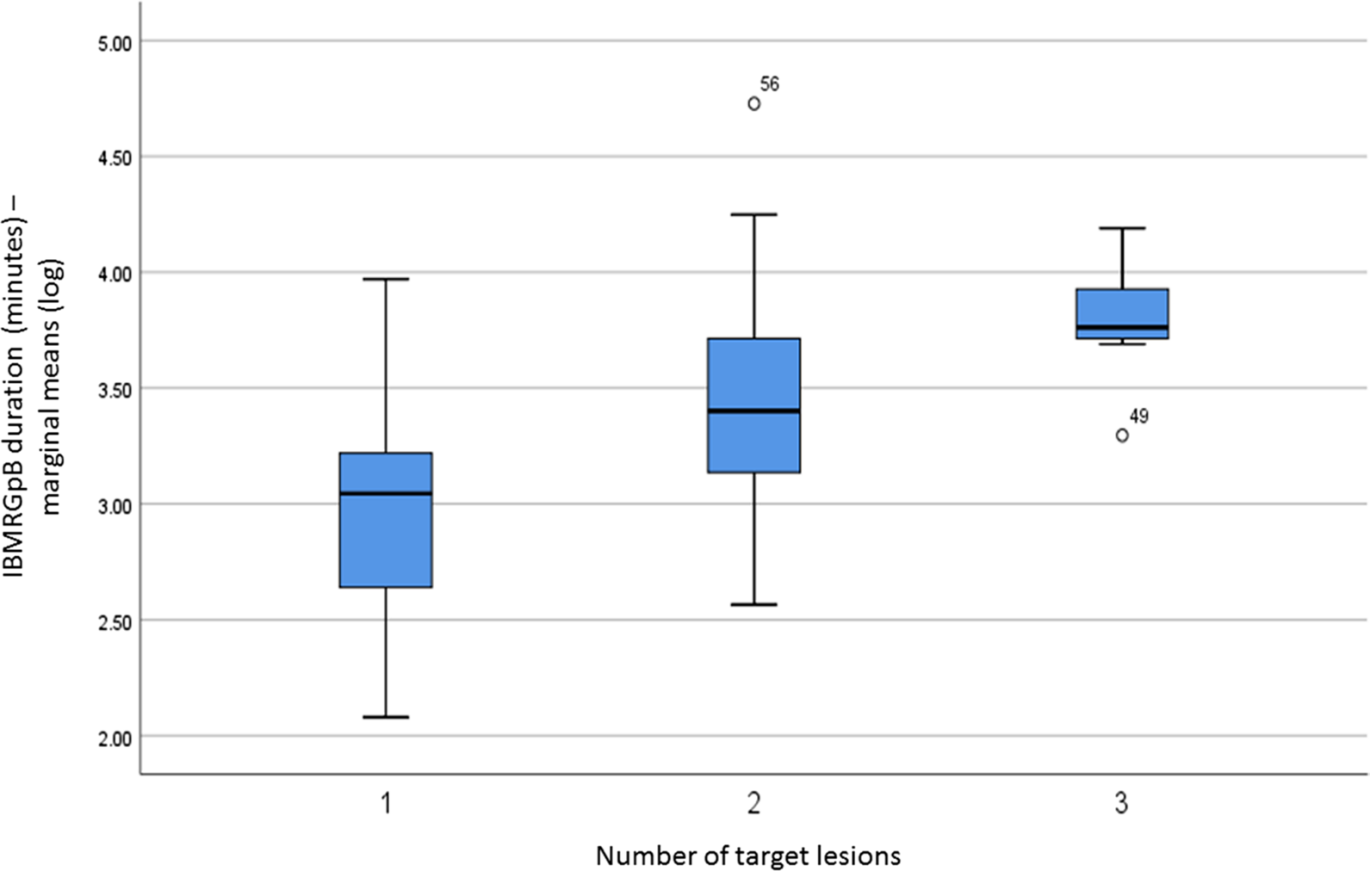
The logarithmic transformation of IB-MRGpB duration (minutes) per target lesion number on MRI imaging. The difference between the sampling time of a single-target lesion compared to that of 2 or 3 target lesions was significant (*p* < 0.0001)

In total, 110 biopsies were evaluated, comprised of 55 for each of the 2 participating urologists. Table 1 lists the patient characteristics and MRI findings. The average time for sampling IB-MRGpB target lesions and per-lesion sampling was 28 ± 15 and 20 ± 10 min, respectively, and there was a gradual decrease in time to completion (per chronological order) (Fig. 1). The number of target lesions correlated with IB-MRGpB-targeted sampling time, showing a significant difference for sampling a single lesion compared to sampling 2 or 3 lesions (*p* < 0.0001) (Fig. 2). Targeted sampling time between 2 and 3 lesions did not differ significantly. Dividing the entire cohort into thirds and evaluating the change in sampling time per lesion needed in the first 18 cases to cases 19–55 revealed a decrease from 37 ± 19 to 24 ± 10 min (*p* < 0.05).

**Table 1:** Patient characteristics and MRI imaging findings

Variable		All		Untrained		Trained		*p* value
Age	Median, IQR	68	62–72	68	62–72	67	62.5–72.5	0.68
PSA (ng/dL)	Mean, STD	8.6	9.1	8.6	9.9	8.7	8.3	0.95
Prostate size (cc)	Mean, STD	53	27	53	27	56	52	0.72
PSA density (ng/dL/cc)	Mean, STD	0.20	0.30	0.22	0.37	0.15	0.10	0.31
Previous biopsy	Number, %	76	70	34	63^*^	42	76	0.13
Number of target lesions on MRI	Mean, STD	1.47	0.6	1.44	0.6	1.51	0.6	0.54
MRI target lesions’ characteristics								
PI-RADS 3	Number, %	84	51.9	38	48.1	46	55.4	0.43
PI-RADS 4	Number, %	62	38.3	34	43.0	28	33.7	0.26
PI-RADS 5	Number, %	16	9.9	7	8.9	9	10.8	0.79
Lesion size (mm)	Median, IQR	7	5–11	7.5	6–11	7	4–12	0.4
Large lesion size (mm)	Mean, STD	10	6	10	6	9	6	0.63
Small lesion size (mm)	Mean, STD	8	6	9	6	7	4	0.095
Lesion location—Base^#^	Number, %	27	17	10	13	17	20	0.2
Lesion location—Mid-gland^#^	Number, %	91	56	39	49	52	63	0.11
Lesion location—Apex^#^	Number, %	56	35	30	38	26	31	0.4
Number of targeted cores/lesion	Mean, STD	3.3	1.6	3.0	1.1	3.6	1.9	0.07

*Untrained* urologist with no experience in performing IB-MRGpBs, *Trained* urologist highly trained in performing IB-MRGpBs, *Large lesion size* represents the size of the larger lesion when more than 1 target lesion was identified on pre-biopsy imaging, *Small lesion size* represents the size of the smaller lesion when more than 1 target lesion was identified on pre-biopsy imaging, *PSA* prostate-specific antigen, *STD* standard deviation, *IQR* interquartile range, *PI-RADS* prostate imaging reporting and data system v.2, *MRI* magnetic resonance imaging

*One patient had no available data regarding former biopsy

^#^Percentages calculated per total number of lesions. Numbers may not add up due to overlap with some lesions located in more than one anatomical section (e.g. base-mid etc.’)

**Fig. 3:**
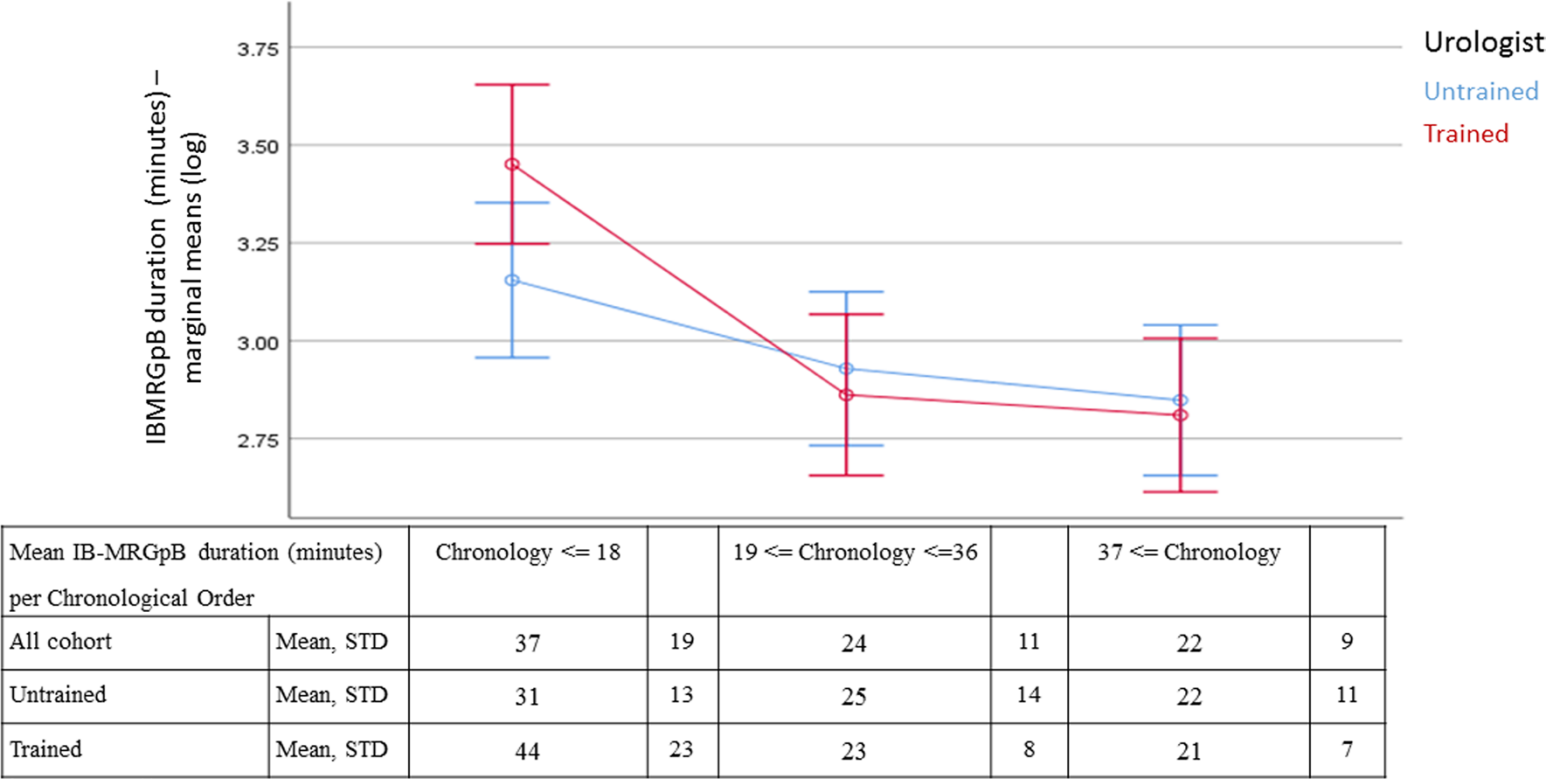
Estimated marginal means of IB-MRGpB time (logarithmic transformation) showing a gradual decrease in procedure time in chronological order of the procedures performed for a single target lesion on MRI. The first 18 cases took longer than the subsequent ones. The table presents the average IB-MRGpB time per single lesion sampling, the procedure time for the entire cohort and the procedure time for the highly trained vs. the untrained urologist. There was no significant difference between the 2 urologists

We evaluated targeted sampling duration per chronological order (i.e., first third of patients vs. second third vs. last third) for each of the urologists separately, controlling for the number of target lesions on MRI (Fig. 3), and then compared their results. The less experienced urologist’s IB-MRGpB targeted sampling time for the first 18 cases (i.e., the first third) showed a trend toward shorter duration (*p* = 0.05). No difference in the IB-MRGpB targeted sampling duration was found between the two urologists for the subsequent two-thirds of patients (from patient number 19 onward). Similar results were found in the analysis of the per urologist IB-MRGpB targeted sampling duration for multiple target lesions (i.e., 2 and 3 lesions) for the first 18 patients compared to the subsequent two-thirds (data not shown).

Neither prostate size, target lesion size (average, maximal, or minimal) nor the usage of diffusion studies during the MRI protocol of IB-MRGpB correlated with the time needed to sample target lesions for the cohort as a whole nor for each urologist’s caseload on separate analyses.

Table 2 lists the correlation between the PI-RADS score with clinically significant disease. A multivariate analysis demonstrated no correlation between positive “hits” (i.e., the finding of clinically significant prostate cancer in correlation with the PI-RADS score) and the following covariates: number of lesions, chronological order of biopsy (i.e., biopsy taken at the first, second, or last third of the cohort) and the operating urologist.

**Table 2:** PI-RADS score correlation with clinically significant disease (“hit”)

PI-RADS score	Clinical significant disease (ISUP ≥ 2)						*p* value
	All	%	Untrained	%	Trained	%	
3	16 (of 84)	19	7 (of 38)	18	9 (of 46)	20	0.89
4	34 (of 62)	55	20 (of 34)	59	14 (of 28)	50	0.49
5	11 (of 16)	69	7 (of 7)	100	4 (of 9)	44	0.74^*^

*PI-RADS* prostate imaging reporting and data system v.2, *ISUP *International Society of Urological Pathology, *Untrained* urologist with no experience in performing IB-MRGpBs, *Trained* urologist highly trained in performing IB-MRGpBs

*A preliminary *p* value of 0.034 was calculated for PIRADS5 lesions. This value was later corrected following Benjamini–Hochberg correction

## Discussion

Multiple techniques are available for performing a biopsy of suspicious prostatic MRI lesions, and we evaluated the urologist’s learning curve of the high-end technique of IB-MRGpB, considered by some to be the leading tool in the diagnosis of clinically significant prostate cancer [1–3, 16]. Our reported correlation between IB-MRGpB targeted sampling and clinically significant disease diagnosis is in agreement with current literature [1, 17]. We achieved a similar level of precision of clinically significant disease diagnosis for smaller lesions as well. For example, the median lesion size was 12 mm (interquartile range [IQR] 8–15) in the PRECISION trial [1], while it was 7 mm (IQR 5–11) in the current work. We believe that the ability to achieve a similar level of biopsy precision for the more challenging smaller lesions represents a real advantage of the IB-MRGpB technique. Our finding is supported by Pokorny et al. [17] who showed that a lesion’s diameter had no impact on cancer diagnosis when using IB-MRGpB.

The most important outcome of prostate biopsy is the ability to detect clinically significant cancer. There was no difference between an experienced urologist’s clinically significant cancer detection rate with that of the less experienced urologist in the present analysis (Table 2). Furthermore, the chronological order of biopsy did not affect the detection rate of clinically significant disease in the entire cohort or in either of the urologists’ cohorts analyzed separately. These data suggest that a urologist’s experience has no effect on the cancer detection rate. El Fegoun et al. and Karam et al. reported similar results for TRUS prostate biopsy and cancer detection rate, supporting the lack of a learning curve regarding cancer detection rates [9, 18]. Lawrentschuk et al. even suggested that it was the operator, rather than the operator’s experience, that influenced the TRUS biopsy cancer detection rate [10]. Acknowledging the aforementioned advantage of MRI-guided prostate biopsy, multiple papers looked at the learning curve of MR/TRUS fusion biopsy and reported that an operator’s learning curve affected sampling accuracy, suggesting that this represents the learning curves of both radiologists and urologists, and that it further affects procedural costs [8, 12, 13]. Similarly, Mager et al. reported that the MR/TRUS fusion biopsy detection rate for the initial 42 cases to be lower compared to the subsequent 42 cases, suggesting that accumulating experience influenced the ability to detect cancer. Of note is the fact that all of the cases reported by those authors were reviewed by a skilled radiologist and with the same supporting team (MRI technician, nurse, and anesthesiologist), suggesting that this learning curve represented the urologist’s improvement rather than that of the rest of the biopsy team members [11].

Despite the application of high-end technology, the IB-MRGpB interface itself is rather simple. The ability to visualize a target lesion traversed by the sampling needle in real time together with a simple operator interface can explain such ease and precision, even at the very early phases of adopting this technology [15]. Our findings of a short learning curve for clinically significant cancer detection suggests an important advantage of the IB-MRGpB over the MR/TRUS fusion biopsy. It should be noted that a comparison of the significant cancer detection rates of PI-RADS 5 lesions between our two urologists’ cohorts might have appeared to reveal a difference in favor of the untrained urologist. Since only very few patients included in this study were diagnosed with PI-RADS 5, however, small diversions can translate into a difference, but this difference did not remain valid after statistical correction.

We perform IB-MRGpB with the patients under general anesthesia. The team consists of technicians, a nurse, radiologist, anesthesiologist, and urologist for ensuring a high standard of care, although it translates into a high procedure cost. As such, the length of a procedure plays a role in preserving resources. The reported procedure time for a transrectal MR-guided biopsy varies from 30 to 68 min [19]. Pokorny et al. [17] estimated the IB-MRGpB execution time to range between 24 and 63 min, and that their own experience averaged 20 min per single target. In line with those figures, our average IB-MRGpB time for sampling of all of the targeted lesions was 28 ± 15 min and the per-lesion sampling duration was 20 ± 15 min, with an average of 3.3 cores sampled per lesion. Importantly, 2 parameters significantly correlated with IB-MRGPB duration, the number of lesions (Fig. 2) and the urologists’ accumulated experience (i.e., chronology) (Fig. 3), representing the procedure’s learning curve. Our findings suggest that the 18th biopsy represents the cutoff point after which procedure time does not improve significantly. Compared to MR/TRUS fusion biopsy, for which the proposed learning curve was 98 cases for targeted biopsy and 84 cases for systematic biopsies in 1 series [13] and 42 cases in another [11], a learning curve of only 18 cases is clearly indicative of a quick and easy-to-adopt technique.

The procedure’s duration plateaued after 18 cases for both urologists. While this may be interpreted as the learning curve of the untrained urologist, shortening the procedure duration over time for the highly trained urologist suggests an alternative explanation. Considering the high level of expertise of all team members to begin with, alongside the fact that they were putting this service together for the first time in our hospital, we believe this to represents the team’s learning curve adopting a synchronized biopsy routine rather than the trained urologists further improvement of skills.

The utilization of MRGpB may be influenced by resource availability and costs, a consideration even suggested by some to specifically limit IB-MRGpB widespread use [20]. While IB-MRGpB indeed necessitates designated team as well as longer MR machine occupancy time, we believe its advantages may overcome these limitations [17, 21]. In the current work we describe how using the IB-MRGpB device and applying high-end technology together with a very simple interface compensates for much of the learning curve needed with other MRI-guided prostate biopsies and allows quick and easy mastering of this technique. In an era in which MRGpB plays such a critical role in prostate cancer diagnosis, such an easy learning curve is an important consideration for choosing a biopsy technique at both the institutional as well as the practicing urologist’s level.

We recognize that our study has several limitations that bear mention. First, we used the PI-RADS correlation with the significant cancer pathology result as surrogate to a positive lesion “hit”. The fact that all MRI reads and IB-MRGpBs were done with the same trained uro-radiologist, and that all specimens were evaluated by a single dedicated expert uropathologist, we believe such consistency and level of expertise translates into true representation of a lesion’s traversing rates. Second, our evaluation of procedure duration included the contribution of multiple personnel, and we extrapolated the urologist’s learning curve from these findings. The fact that all other team members were highly experienced to begin with supports this approach. Lastly, our data is limited by the small cohort size, however, we believe the significant findings we show can serve the urologic-radiologic community.

In conclusion, these data suggest a very short learning curve for IB-MRGpB-targeted sampling duration, and that clinically significant cancer detection rates are not influenced by the learning curve of this technique.

## Data Availability

All of the material is owned by the authors and will be delivered upon request. Please contact Barak Rosenzweig MD at Barak.rosenzweig@sheba.health.gov.il; barak22@gmail.com.

## Abbreviations

mpMRI: Multi-parametric magnetic resonance imaging
MRGpB: MRI-guided prostate biopsy
IB-MRGpB: In-bore MRI-guided prostate biopsy
TRUS: Transrectal ultrasound
PI-RADS: Prostate imaging reporting and data system
